# Tetracycline-controlled (TetON) gene expression system for the smut fungus *Ustilago maydis*

**DOI:** 10.3389/ffunb.2022.1029114

**Published:** 2022-10-19

**Authors:** Kishor D. Ingole, Nithya Nagarajan, Simon Uhse, Caterina Giannini, Armin Djamei

**Affiliations:** ^1^ Department of Plant Pathology, Institute of Crop Science and Resource Conservation (INRES), University of Bonn, Bonn, Germany; ^2^ Austrian Academy of Sciences (OEAW), Vienna Biocentre (VBC), Gregor Mendel Institute (GMI), Vienna, Austria

**Keywords:** *Ustilago maydis*, smut disease, tetracycline, TetON, transcriptional inducible system, Bax1, toxic protein

## Abstract

*Ustilago maydis* is a biotrophic phytopathogenic fungus that causes corn smut disease. As a well-established model system, *U. maydis* is genetically fully accessible with large omics datasets available and subject to various biological questions ranging from DNA-repair, RNA-transport, and protein secretion to disease biology. For many genetic approaches, tight control of transgene regulation is important. Here we established an optimised version of the Tetracycline-ON (TetON) system for *U. maydis*. We demonstrate the Tetracycline concentration-dependent expression of fluorescent protein transgenes and the system’s suitability for the induced expression of the toxic protein *BCL2 Associated X-1 (Bax1)*. The Golden Gate compatible vector system contains a native minimal promoter from the *mating factor a-1* encoding gene, *mfa* with ten copies of the tet-regulated operator (tetO) and a codon optimised Tet-repressor (tetR*) which is translationally fused to the native transcriptional corepressor Mql1 (UMAG_05501). The metabolism-independent transcriptional regulator system is functional both, in liquid culture as well as on solid media in the presence of the inducer and can become a useful tool for toxin-antitoxin studies, identification of antifungal proteins, and to study functions of toxic gene products in *Ustilago maydis*.

## Introduction

The ability to regulate gene expression at will is a valuable genetic approach to study gene functions (Lewandoski, 2002). To turn-ON or -OFF gene expression at the desired time reduces the secondary effects of the gene product and hence can lead depending on the expression time to the identification of its primary functional role in the cell. Heterologous regulatory systems are useful tools for controlled gene expression since they rarely interfere with endogenous metabolic regulation. One such versatile heterologous component system is the tetracycline (Tet)-regulated expression system. Tet-regulated systems are often used to study functions of genes *in vivo* by regulating their activity quantitatively, reversibly and in a temporally defined way to observe resulting phenotypic changes (Gossen et al., 1995). This system originates from the tetracycline-resistance operon of gram-negative bacteria, in which it controls the expression of the antiporter, called *TetA* which pumps out the antibiotic tetracycline, driven by the simultaneous uptake of protons (H^+^) into the cell (Berens and Hillen, 2004). Tet-regulated expression systems have been adapted in many eukaryotic organisms including many vertebrates and non-vertebrate organisms, plants, unicellular organisms, and culture cell lines. However, while adapting this heterologous system to eukaryotes, various modifications have been made to adapt the system to different organisms (Weinmann et al., 1994; Gossen and Bujard, 2002; Berens and Hillen, 2004). Antibiotics of the tetracycline family such as tetracycline (Tet) or doxycycline (Dox) are membrane-permeable and most importantly lack known targets in eukaryotic cells (Wishart et al., 2005).

There are two different Tet-regulated promoter systems; one called TetOFF and another called TetON system. For the first system, tetracycline is administered to switch off the transcription. This is achieved by causing interference with transcriptional initiation by a Tet-dependent trans-activator (called tTA) which was generated by fusing 207-amino acid (207-aa) of Tet repressor (TetR) to the 127-aa transcriptional activation domain (AD) of the viral protein 16 (VP16) from Herpes Simplex virus (Triezenberg et al., 1988). TetR and its cognate operator element *tetO* naturally exhibit very high affinity. The tTA gets off upon Tet-binding from the promoter and thereby transcriptional initiation is stopped upon tetracyclines added to the medium (Brent and Ptashne, 1985). A hybrid promoter consisting of 7 copies of *tetO* binding sites upstream of an eukaryotic basal promoter is used to regulate tTA dependent expression (Gossen and Bujard, 2002; Berens and Hillen, 2004). In absence of Tet or Dox, tTA dimers will bind the *tetO* sites and activate the expression of the downstream gene (Gossen and Bujard, 1992; Das et al., 2016).

**Figure 1:**
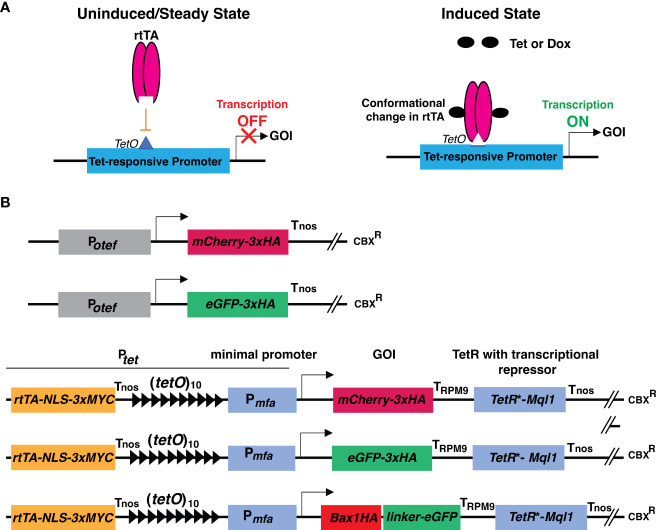
Schematic representation of principle of TetON system and cloning strategies used in this study. **(A)** Schematic representation showing the operational principle of the TetON system in uninduced and induced conditions. **(B)** Schematic representations of constructs with P*_otef_* and P*_tet_* used in this study. Arrow indicates the transcriptional start site.

The disadvantage of the TetOFF system is, that Tet or Dox has to be added continuously to stop the expression of the Gene of interest (GOI), and also Tet has to be completely removed from the medium to resume gene expression, hence transient expression studies were cumbersome. To overcome these problems, the TetON system was developed (Gossen et al., 1995). TetON system allows activation of gene expression by administration of Tet or Dox to the medium (**Figure 1A**). For the development of the TetON system, a mutated version of TetR* was generated which binds to *tetO* only in the presence of Tet or Dox due to change in its conformation. Fusion of this mutated TetR* to VP16 activation domain generated reverse transactivator-tTA (called rtTA) which binds to *tetO* and activates transcription only in presence of the antibiotic (Gossen et al., 1995) (**Figure 1A**).

Previously efforts were made to adapt the Tet-regulatory transcriptional system for the phytopathogenic fungus *U. maydis*. In *U. maydis*, the non-codon optimized *TetR* gene showed premature polyadenylation and also the transcriptional activation domain of VP16 seemed to be toxic to *U. maydis* cells. These problems were overcome by generating a codon-optimized synthetic *tetR* ^∗^ gene, removing cryptic enhancer elements from the promoter and using an acidic minimal activation domain. Authors showed the abolishment of mating on solid media by administration of Tet to suppress the expression of pheromone response factor, *Prf1* (Zarnack et al., 2006). This resulted in generation of TetOFF system for *U. maydis* however a tight TetON system was not available for gene expression studies in *U. maydis*.

Therefore, in this study, we describe the optimised and functional TetON system for *U. maydis*. Basal leaky expression is the most critical and common problem with many inducible gene expression systems. To overcome this, we used Mql1 (UMAG_05501) from *U. maydis* in fusion to TetR^∗^. Mql1 is the closest ortholog of the transcriptional co-repressor Ssn6 from yeast. The Tup1-Ssn6 complex from budding yeast is one of the best studied corepressors in eukaryotes. Tup1-Ssn6 represses subsets of genes when recruited to their promoters by a sequence-specific manner. This transcriptional repression involves interactions among the corepressor and hypoacetylated histones, histone deacetylases, and the RNA transcriptional machinery (Malavé and Dent, 2006).

We first demonstrate the tightly regulated expression of fluorescent proteins encoding genes (mCherry and eGFP) using the TetON system. Confocal microscopy revealed, that these fluorescent proteins were highly induced upon addition of Tet to potato dextrose agar (PDA) plates as well as in axenic cultures. The induction occurs in a dose-dependent manner starting from non-detectable amounts prior Tet addition. Beside fluorescent microscopy the inducible expression of mCherry and eGFP was verified by immunoblotting. As a proof of concept, we tested the expression of the toxic gene *Bax1* using the modified TetON system and observed that Bax1-HA-linker-eGFP fusion protein was only induced after administration of Tet to the medium. The growth inhibitory effect of Bax1 was observed on solid PDA medium. Similarly, Bax1 expressing cells showed attenuated growth in liquid cultures after 1 dpi which was documented by growth curve analysis. In the uninduced state, confocal microscopy and immunoblotting results revealed that neither fluorescent proteins (mCherry and eGFP) nor Bax1-HA-eGFP showed expression indicating that the adapted TetON system is not leaky and operates under very tight control by Tet administration.

## Materials and methods

### Plasmids and cloning procedures

*Escherichia coli* Mach1 T1 phage-resistant (T1R) (Thermo Fisher Scientific, Waltham, MS, USA) chemical competent cells were used for cloning purposes. The plasmids used in this study were generated using either standard molecular cloning procedures (Sambrook et al., 1989) or by using the GreenGate Cloning system (Lampropoulos et al., 2013). Plasmids used in this study are described in **Supplementary Table 1**. The modules used were either amplified by PCR or obtained from the published GreenGate Cloning system (Lampropoulos et al., 2013). High fidelity proofreading DNA polymerase (*Phusion polymerase*, New England Biolabs) was used for PCR amplification to generate desired DNA modules. Restriction enzymes were purchased from New England Biolabs (Ipswich, MA, USA). Primers used and their purposes are listed in **Supplementary Table 2**.

### Construct design

The TetON vector consists of a reverse tetracycline-transactivator 3 (rtTA3) operated by the promoter (433 bp upstream of the start codon) of the gene UMAG_05521 (belongs to the hypothetical sugar phosphate phosphatase family) which has constitutive expression. Nuclear localization signal (NLS) with triple MYC epitope was fused to the C-terminal region of rtTA3, followed by the nopaline synthase terminator (Tnos) (Bevan et al., 1983). The 339 bp minimal promoter from pheromone gene *mfa* (P*_mfa_*) along with 10 copies of tetracycline operators (*tetO*) were used to drive the expression of the gene of interest (*mCherry/eGFP/Bax1*) tailed by the terminator of the Pheromone Regulated Membrane protein (PRM9) from *Saccharomyces cerevisiae* (Curran et al., 2013). The Tet-repressor (TetR*) was codon optimized for *U. maydis* and its expression was driven by the promoter of the gene UMAG_05521. The transcriptional repressor *Ssn6* (CYC8) ortholog *Mql1* from *U. maydis* (UMAG_05501) with six tetratricopeptide repeat (TPR) domains was translationally fused to the TetR* tailed by the Nos terminator (**Figure 1B** and **Supplementary Figures 3**, **4**). The whole cassette was assembled in a modified Golden Gate compatible p123 vector (Loubradou et al., 2001; Navarrete et al., 2022).

### Generation of *U. maydis* strains and fungal growth conditions

All *U. maydis* strains from this study are derived from the solopathogenic strain SG200 (a1, mfa2, bE1/bW2; (Bölker et al., 1995). Protoplast preparation and transformation of *U. maydis* strain SG200 was carried out as previously described (Bösch et al., 2016). Briefly, for each construct, around 5μg of the plasmid DNA was linearized by digestion with restriction enzyme *Ssp*I, and *U. maydis* protoplasts were transformed with linearized DNA for the integration into the *ip* locus by homologous recombination using *p*123 derivatives as described previously (Loubradou et al., 2001; Navarrete et al., 2022). The individual colonies were grown on Potato Dextrose Agar (PDA) (Merck, Kenilworth, NJ, USA) media with respective antibiotics (Carboxin, CBX 2μg/ml). The transformants were confirmed for the presence of the desired insert by PCR. All the *U. maydis* strains were grown at 28°C in YEPS liquid medium (0.4% yeast extract, 0.4% peptone, and 2% glucose) with overnight shaking at 180 rpm.

### TetON induction test in solid and liquid media

Around 1ml of one day grown culture of *U. maydis* strains were pelleted by centrifugation at 3500 rpm for 5 minutes and washed once with 1ml sterile water. Then the cells were resuspended in sterile water to OD_600_ = 1.0. For solid media, around 5μl of the culture was spotted on the PDA plate with carboxin (2μg/ml) and different tetracycline concentrations (2, 5, 10, 20, 50, and 100 μg/ml). Four days grown fungal sporidia were dissolved in sterile water and around 2μl of the suspension was observed under the confocal microscope. For testing Bax1 growth inhibition effect by plating assay, 100 μl of OD_600_ = 0.2 and serial dilutions of 1:10, 1:100 and 1:1000 were plated on PDA plate ± Tet (50 μg/ml), pictures were taken after 3^rd^ and 5^th^ day of incubation and colony forming units (CFU) were counted at day 3.

For induction in liquid culture, the strains were grown in 5ml of the YEPS liquid media containing respective antibiotics and tetracycline concentration of 100μg/ml (based on the results from the solid agar plate). After 24 hours, around 2μl of the cell culture was placed on a glass slide and confocal microscopy was performed.

### TetON induction test in infected maize plants

The sweet corn seeds of Early Golden Bantam (EGB) were germinated in pots filled with vermiculite. Seven days old seedlings were infected with SG200 and SG200 carrying *P_otef/tet_-mCherry-3xHA* and *P_otef/tet_-eGFP-3xHA* with OD_600_ = 1. After 48 hrs post infection, plants were supplied with ¼ strength of Murashige and Skoog (MS) liquid medium ± Tet (100 ug/ml), and confocal microscopy was performed after 24 hrs post Tet administration.

### Immunoblotting and confocal microscopy

After 5-dpi, an equal amount of fungal sporidial growth was collected from the induction plate for immunoblotting. Briefly, the fungal sporidia were dissolved in 200 μl of 0.2M NaOH by vortexing along with 50mg sand particles for cell lysis. The appropriate amount of 4x Laemmli protein sample buffer (100mM Tris, 2% SDS, 2mM EDTA, 0.01% Bromophenol blue, 20% Glycerol, 50mM DTT) was added and the samples were boiled at 95°C for 10 minutes to break the disulfide bridges and denature the samples. The supernatant was collected after centrifugation at 13500rpm for 1 minute and 10 μl was loaded to SDS-PAGE for immunoblotting with α-HA (Sigma-Aldrich, St. Louis, MO, USA), α-GFP (BIOZOL/MBL-598), α-MYC (Sigma-Aldrich, St. Louis, MO, USA) and α-Actin (Invitrogen, Waltham, MA, USA) antibodies.

Confocal microscopy was performed with TCS SP8 confocal microscope (Leica, Germany) and processed with Leica Application suite (LASX). Argon laser at 488 nm was used for eGFP with excitation and the emission of 485nm and 535 nm respectively. For mCherry, the excitation was at 561 nm, and emission was recorded between 578 and 648 nm.

### Growth curve assay

The *U. maydis* strains were inoculated in 20ml of YEPS liquid media in 100ml baffled flasks with respective antibiotics. One day old culture was used as pre-inoculum and the initial cell density was set as OD_600_ = 0.2 and incubated at 28°C with shaking at 180 rpm. The OD_600_ was measured after 2, 4, 6, 10, and 20 hours post-induction (hpi). For plating assay, overnight grown cultures were harvested and 100 μl cells with OD_600_ = 0.2 were plated on PDA ± Tet (50 μg/ml). Colonies were counted at day 3 and phenotypic pictures were taken on day 3 and 5. The experiments were repeated twice with similar conditions.

### Filamentation test

The TetON strains were tested for their ability to form infectious filamentous structures by growing them on PDA-charcoal plates (PDA with 1% Charcoal). Briefly, the strains were grown in YEPS liquid media until the exponential phase (OD_600_ = 0.6-0.8) and harvested by centrifugation at 3500 rpm for 5 minutes. The cells were washed once with sterile ddH_2_O and cells with OD_600_ = 1.0 were spotted on PDA-Charcoal plates.

## Results

### TetON system induces expression of neutral fluorescent genes only in presence of the antibiotic Tetracycline

To test the expression of neutral genes, we cloned mCherry-3xHA and eGFP-3xHA under the control of a Tet-regulated promoter (*P_tet_*). In brief, we used 10 copies of Tet operator elements [(*tetO*)_10_] followed by the minimal promoter from the pheromone gene *mfa1* (P*mfa1*) of *U. maydis* (Bölker et al., 1992; Spellig et al., 1994). The genes of interest, *mCherry*, and *eGFP* were tagged with 3xHA (Hemagglutinin) epitope at the C-terminus. The PRM9 terminator (T_PRM9_) from yeast was used for transcriptional termination. To avoid the expression under uninduced conditions, codon-optimized TetR* was translationally fused with the transcriptional corepressor *Mql1* of *U. maydis* (*Ssn6* ortholog of *U. maydis*) harboring six tetratrico peptide repeat region (TPRs) with T_nos_, as a transcriptional terminator of the nopaline synthase gene derived from *Agrobacterium tumefaciens* (Bevan et al., 1983) as shown in **Figure 1B**; **Supplementary Figures 3, 4**.

**Figure 2:**
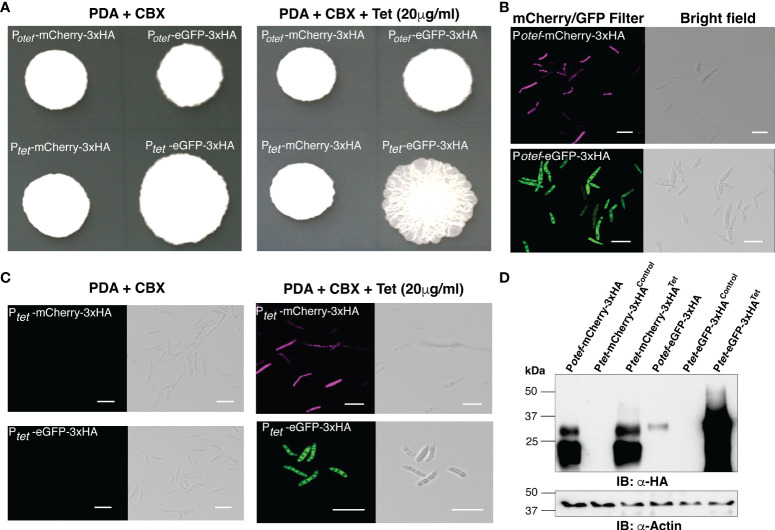
Induction of mCherry and eGFP with TetON system. **(A)** Growth of indicated *U. maydis* strains on Potato Dextrose agar (PDA)+CBX ± Tet (20 μg/ml). Plate photographs were taken on the 4th day after spotting. **(B)** Confocal images of cells from SG200:P*_otef_*-mCherry-3xHA and SG200:P*_otef_*-eGFP-3xHA after 4 days of growth on PDA plates (Scale bar= 20 μm). **(C)** Confocal images of cells from SG200:P*_tet_*-mCherry-3xHA and SG200:P*_tet_*-eGFP-3xHA after 4 days of growth on PDA+CBX ± Tet (20 μg/ml) plates (Scale bar= 20 μm). **(D)** Immunoblot with α-HA and α -Actin antibodies (loading control) on total protein extracts from indicated strains grown on PDA+CBX ± Tet (20 μg/ml) plates.

*U. maydis* solo-pathogenic positive control strains SG200:P*_otef_-*mCherry-3HA, SG200:P*_otef_*-eGFP-3xHA and the test-strains SG200:P*_tet_-*mCherry-3xHA and SG200:P*_tet_*-eGFP-3xHA were initially grown in liquid cultures with carboxin (CBX, 2 μg/ml) in 28°C shaker for overnight. The washed cells of Optical Density at 600nm (OD_600_) 1.0 were spotted on PDA+CBX ± Tet (20 μg/ml) plates and incubated at 28°C for 4 days (**Figure 2A**). All tested strains grew normally in presence of Tet and also showed normal filamentation on the PDA+charcoal medium (**Supplementary Figure 1**). Taken together, this data indicates that the antibiotic Tet does not have any adverse effect on the growth of *U. maydis* in axenic culture. Further to visualize reporter protein induction, we performed confocal microscopy on all these strains and observed that the mCherry and eGFP fluorescent proteins were expressed in the positive control strains P*_otef_* constitutively. However, P*_tet_* induced the expression of mCherry/eGFP only upon Tet administration (**Figures 2B, C**). To validate these results, we also performed an immunoblot with anti-HA antibodies on total protein extracts and detected the same pattern of expression. The mCherry-3xHA expression with P*_tet_* was comparable to the level of mCherry-3xHA expression driven by the strong constitutive P*_otef_* promoter whereas we observed very high expression of eGFP-3xHA with P*_tet_*, higher than with P*_otef_* (**Figure 2D**). We also tested the expression of rtTA-3xMYC (expected size is ~42 kDa) by performing immunoblot on total protein extracts from SG200 harboring P*_otef-_* eGFP (as negative control) and P*_tet-_* eGFP constructs with anti-MYC antibodies (**Supplementary Figure 1B**). Taken together, the expression of mCherry/eGFP with P*_tet_* promoter is only detectable in the presence of Tet.

### Tetracycline-regulated expression of *Bax1* induces growth inhibition in *U. maydis*

**Figure 3:**
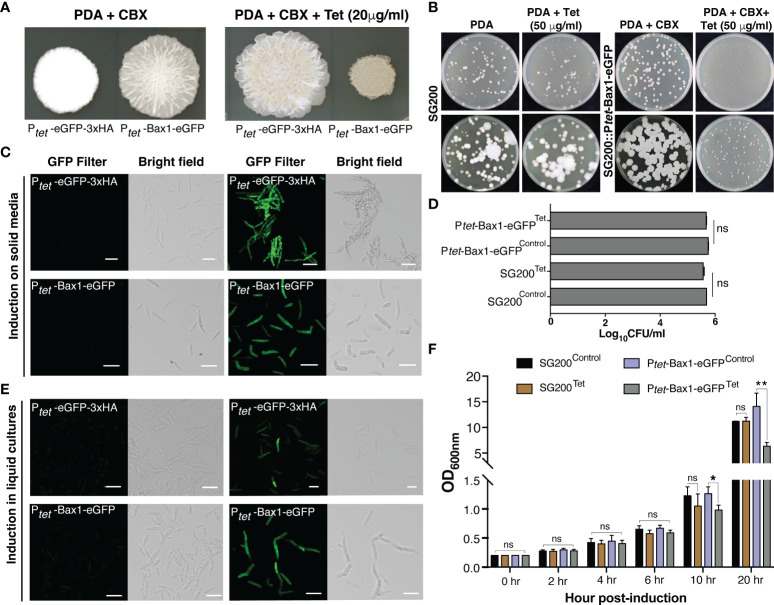
Tetracycline regulated expression of toxic gene Bax1 in *U. maydis*. **(A)** Growth of indicated *U. maydis* strains on PDA+CBX ± Tet (20 μg/ml) plates after day 4 of spotting. **(B)** The phenotype of SG200 and SG200::P*_tet_*-Bax1-eGFP colonies on PDA ± Tet (50 μg/ml) induction at 3^rd^ and 5^th^ day. **(C)** Confocal images of cells from SG200:P*_tet_*-eGFP-3xHA and SG200:P*_tet_*-Bax1-HA-eGFP after 4 days of growth on PDA+CBX ± Tet (20 μg/ml) plates. **(D)** Graph showing CFU/ml counted from **(B)**. **(E)** Confocal images of cells from one day grown liquid uninduced and induced cultures of SG200:P*_tet_*-eGFP-3xHAand SG200:P*_tet_*-Bax1-HA-eGFP (Scale bar in all images = 20 μm). **(F)** Growth curve (OD_600_ graph) of *U. maydis* cultures of indicated strains ± Tet (100 μg/ml), X-axis indicates time points as hpi, and Y-axis indicates OD_600._ Significant differences were determined using Student’s t-test (*p < 0.05, **P < 0.01; ns, not significant).

As a proof of principle, we tested Tetracycline regulated expression of the toxic gene, *Bax1* in *U. maydis* on PDA plate since its expression induces mitochondrial apoptosis followed by cell death in yeast *S. cerevisiae* (Hanada et al., 1995; Wang et al., 1996; Cheng et al., 1997; Khaled et al., 1999). Surprisingly, induction of Bax1 in the strain SG200:*P_tet_*-Bax1-HA-linker-GFP with 20 μg/ml Tet on a PDA plate showed reduced growth compared to growth on an uninduced plate (**Figures 3A, B**). Confocal microscopy of the SG200 strain bearing *P_tet_-Bax1-HA-linker-GFP* without Tet administration (on PDA + Carboxin containing plate) did not show GFP fluorescence which ensured that this system is not leaky. Administration of 20 μg/ml Tet to PDAplates induced the expression of Bax1-HA-eGFP as shown in **Figure 3C**. The expression of eGFP alone was also tested as a control which was observed as expected. With these results, we conclude that the induction of fluorescent proteins, as well as toxic protein, are highly specific to Tet addition to solid PDA plates.

To test whether regulated gene expression using the TetON system can also be performed in axenic cultures, we grew *SG200:P_tet_-eGFP-3xHA* and *SG200:P_tet_-Bax1-HA-eGFP* in YEPS liquid media overnight and then started secondary cultures with OD_600_ = 0.2. We added 100 μg/ml Tet for induction and performed confocal microscopy 24 hours post induction (hpi). Both neutral proteins, eGFP as well as the supposedly toxic protein Bax1-HA-eGFP were expressed in liquid cultures induced with Tet. We did not observe GFP fluorescence in uninduced cultures indicating that gene expression only happened upon administration of the inducer molecule Tet. To quantify the growth inhibitory effect of Bax1 expression in liquid cultures, we measured the growth curve of *SG200* progenitor and *SG200:P_tet_-Bax-1HA-eGFP* strains. These strains were initially grown in YEPS liquid with appropriate antibiotic selection and then secondary cultures were inoculated with OD_600_ = 0.2 ± Tet 100 μg/ml concentration. The OD_600_ was measured at 0, 2, 4, 6, 10, and 20 hpi. As expected, Bax1 induced cultures showed a statistically significant difference in the OD_600_ at 10 and 20 hpi time points (**Figure 3F**) indicating that Bax1 expression interferes with the growth rate of *U. maydis* cells. Dead cells or cell debris can interfere with OD_600_ measurements and hence to test the growth inhibitory effects of Bax1 induction we performed a plating assay. For this, progenitor strain SG200 and the strain carrying *P_tet_-Bax-1HA-eGFP* were grown overnight in YEPS liquid cultures. Cells with OD_600_ = 0.2 and 1:10 dilutions were plated on PDA *±* Tet (50 μg/ml). The pictures were taken at three and five days after incubation at 28°C, and cell counting was also performed. Cells were growing slower when Bax1 was induced with Tet addition compared to uninduced plate however unexpectedly there were no differences in colony forming units (CFU) from induced and uninduced plates (**Figures 3B, D**). This implicates that unlike in the ascomycete *S. cerevisiae*, Bax1 is not leading to programmed cell-death in the basidiomycete *U. maydis* but to a significant growth retardation of the cells.

### TetON system operates in a concentration-dependent manner

**Figure 4:**
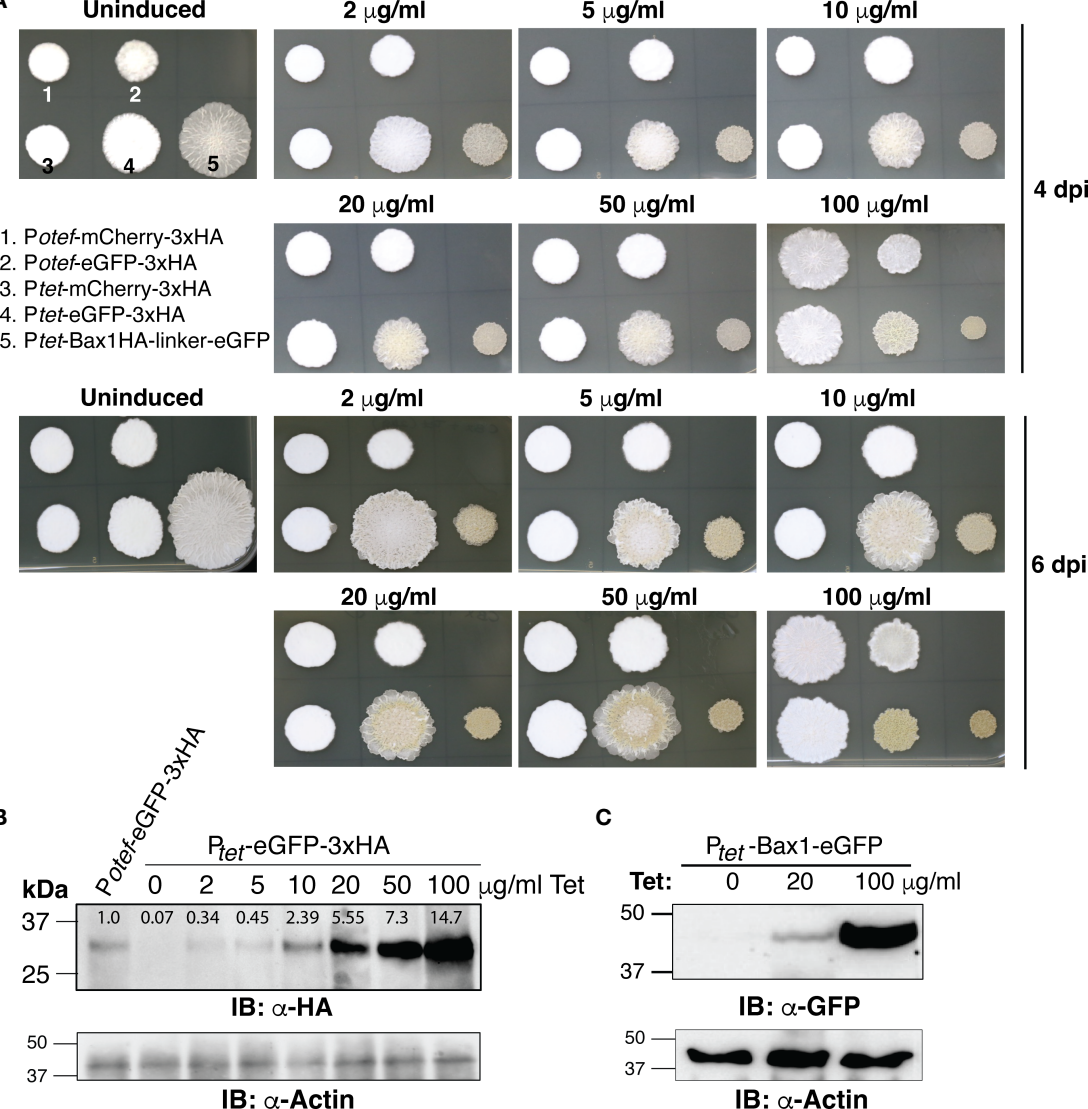
Dose-dependent induction of gene expression by TetON system. **(A)** The phenotype of fungal growth on the 4^th^ and 7^th^ day of indicated *U. maydis* strains grown on PDA+CBX ± Tet (2, 5, 10, 20, 50, and 100 μg/ml). **(B, C)** Immunoblot performed with α-HA, α-GFP, and α-Actin antibodies on total protein extracts of sporidia harvested from 4 days old, indicated strains.

To test whether the TetON system shows concentration-dependent gene expression and in which range, we spotted OD_600_ = 1.0 of SG200 harboring *P_otef_-mCherry-3HA, P_otef_-eGFP-3HA, P_tet_-mCherry-3HA, P_tet_-eGFP-3xHA* and *P_tet_-Bax1-HA-linker-eGFP* on PDA+CBX plates containing 0, 2, 5, 10, 20, 50 and 100 μg/ml Tet concentrations. As shown in **Figure 4A**, Bax1 induction causes a growth inhibitory effect even with 2 μg/ml Tet concentration and the effect gradually increased with Tet concentration. Also, the eGFP expression with the P*_tet_* promoter was stronger than the constitutive *P_otef_* promoter. This was also evident with the visual appearance of a greenish color of the sporidial growth of SG200*:P_tet_-*eGFP-3HA. To validate this result, we performed immunoblot with anti-HA and anti-GFP antibodies on total protein extracts from sporidial growth harvested from SG200*:P_tet_-*eGFP-3xHA and SG200*:P_tet_-*Bax1-eGFP colonies grown on PDA+CBX plates with different Tet concentrations. Immunoblotting confirmed the gradual increase in expression of eGFP starting from 2 μg/ml to maximum expression at 100 μg/ml (**Figure 4B**). We also measured the intensity of eGFP-HA bands from **Figure 4B** with ImageJ (https://ij.imjoy.io/) software and calculated the correlation coefficient (R^2^-value). An R^2^ of 0.9645 indicates that the regression predictions perfectly fit with the data (positive correlation between Tet concentration and eGFP-HA expression levels). The Bax1-eGFP protein expression was also detectable from sporidial growth with 20 μg/ml Tet concentration and maximum expression was observed at 100 μg/ml (**Figure 4C**). The expression of eGFP with *P_tet_* promoter is several folds higher than that of eGFP expression with P*_otef_* promoter (**Figure 4B**). Our data strongly supported the notion that the TetON system operates in a concentration-dependent manner in a wide range of genes encoding both, neutral and toxic gene products.

### *In-planta* testing of TetON system

To test whether the TetON system we generated can be used for induction of gene expression in *U. maydis* during its biotrophic interaction phase with host plant-maize, we grew maize seedlings in pots filled with vermiculite. Seven days old seedlings were infected with progenitor strain SG200 and SG200 carrying *P_otef/tet_-mCherry-3xHA* and *P_otef/tet_-eGFP-3xHA* with OD_600_ = 1. After 48 hrs post infection, plants were supplied with ¼ strength of Murashige and Skoog (MS) liquid medium ± Tet (100 μg/ml), and confocal microscopy was performed after 24 hrs post Tet administration to check the induction of reporter genes- mCherry and eGFP.

**Figure 5:**
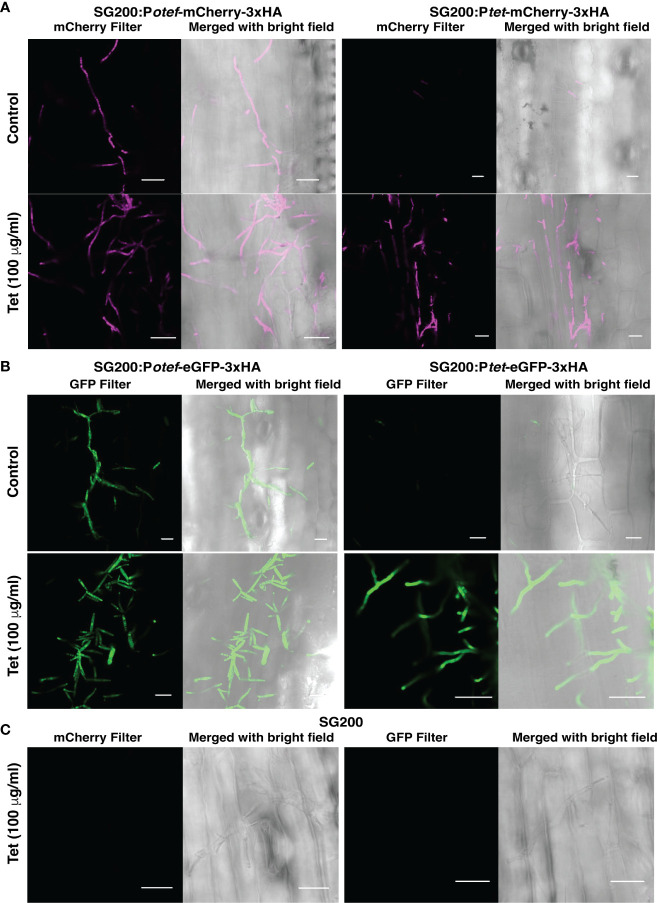
Tet-regulated expression of mCherry and eGFP in infected maize plants. **(A)** Expression of mCherry with P*_otef_* and P*_tet_* promoter in control and Tet-added maize plants at 3 dpi. **(B)** Expression of eGFP with P*_otef_* and P*_tet_* promoter in control and Tet-added maize plants at 3 dpi. **(C)** Maize infected cells showing hyphal growth of progenitor strain SG200 at 3 dpi in Tet-added condition (Scale bar = 20 μm).

As expected, maize plants infected with SG200 expressing mCherry and eGFP under *otef* promoter showed fluorescent signal in control as well as Tet-added conditions which confirmed that the expression is constitutive whereas maize plants infected with SG200 carrying P*_tet_*-mCherry-3xHA and P*_tet_*-eGFP-3xHA strains showed respective fluorescent signal only in plants administered with Tet (**Figures 5A, B**). The maize plants infected with progenitor strain SG200 did not show fluorescence under mCherry/GFP filter (**Figure 5C**).

## Discussion

Synthetic biology has emerged as a versatile discipline based on rational and synthetic approaches to study and modify biological systems for the specific needs of humans (Benner and Sismour, 2005). The success of synthetic biology tools depends on techniques that can combine gene network elements in a modular fashion to create complex genetic architectures with the desired usability. Tet-regulated gene expression systems have been adapted to several ascomycetes such as *Saccharomyces cerevisiae* and *Candida albicans* (Garí et al., 1997; Park and Morschhäuser, 2005). Tet-system has also been used to activate silent gene clusters in the notorious rice pathogen *Fusarium fujikuroi* (Janevska et al., 2017).

For gene expression studies in *U. maydis*, various native constitutive promoters like P*_otef_* and P*_oma_* are available which are active during the saprophytic growth phase (Sarkari et al., 2014; Zambanini et al., 2017) whereas native inducible promoters like P*_tad1_*, P*_mtt1_*, P*_crg1_* P*_nar1_* and P*_shy1_* are also available (Bottin et al., 1996; Brachmann et al., 2001; Rabe et al., 2013; Zambanini et al., 2017) which are induced/repressed by application of arabinose/nitrate/ammonium/glucose/Salicylic acid (SA) molecules respectively. Biotrophy-specific promoters like P*_Cmu1_* and P*_pit2_* (Djamei et al., 2011; Doehlemann et al., 2011) are also existing to study effector functions. However, most of these native promoters are linked to basic metabolic processes of fungus either during saprophytic growth or biotrophic growth cycle and therefore induction or repression of gene expression by using these promoters would interfere with fungal metabolism by unknown mechanisms. Therefore, for precise gene functional studies, it is advisable to develop a gene expression system that does not interfere with fungal metabolism and can be operated from outside. Towards this, here we report one such synthetic titratable gene expression tool for the phytopathogenic smut fungus *U. maydis* by acquiring non-endogenous as well as endogenous genetic elements and we have built a cassette which turns-on gene expression upon administration of the antibiotic Tet.

We used synthetic codon-optimized tetR* gene for *U. maydis* which prevented premature polyadenylation (Zarnack et al., 2006). Earlier studies showed that the minimal activation domain (AD) from VP16 expressed with constitutively active P*_otef_* promoter was toxic to *U. maydis* cells possibly because of squelching of the transcriptional machinery, hence in our study, we used the minimal promoter from the endogenous pheromone gene, *mfa1* (denoted as P*_mfa_* in **Figure 1B**) (Gill and Ptashne, 1988; Spellig et al., 1994). One of the critical problems of inducible gene expression systems is the leakiness of expression. To minimize this, one important modification was the use of a native transcriptional corepressor, the Ssn6 ortholog Mql1 of *U. maydis*. Surprisingly, we observed that supposedly toxic proteins like Barnase (Hartley, 1988; Mireau et al., 2003; Edelweiss et al., 2008) showed no toxic effect upon induction. in *U. maydis* (**Supplementary Figure 2**). One explanation could be, that despite the fact, that we could not detect in western blot under uninduced conditions expression of the transgene, residual leakiness might lead to negative selection of expressing strains or to compensatory effects in the metabolism that minimize toxicity. Also, *U. maydis* expressing eGFP-3xHA (P*_tet_*-GFP-3xHA) affects colony morphology which might be potential photo-toxicity caused by reactive species generated by the very high levels of GFP (Ganini et al., 2017) (**Figure 2A**). Already under non inducing conditions the colony appearance of P*_tet_*-Bax1-eGFP expressing strains is affected indicating potentially leaky expression of eGFP-Bax1 under uninduced condition although beyond the detection limit of immunoblotting (**Figure 4C**) and confocal microscopy (**Figures 3C, E**). However, in the case of Bax1, we observed a clear dose-dependency on growth inhibition. Surprisingly the plating assay of dilutions on Tet versus control plates indicates that not cell death but only growth retardation is caused by Bax1 overexpression in *U. maydis*. Furthermore, the used fusion proteins (linker-eGFP tag) had a decisive influence on the observed or not observed toxicity in the case of Bax1 (**Supplementary Figure 2**) which could be connected with the functional folding and stability of the fusion protein. The molecular events leading to these phenomena are beyond the scope of this technical paper and would require separate attention.

The usage of the Golden Gate based modular cloning system (Lampropoulos et al., 2013) allows a large versatility of combinations and constructs to be tested to optimize individually the inducible expression of proteins with respective N or C-terminal tags or by combining the expression of two proteins under the same TetON system *via* a P2A ribosomal skipping motif (Müntjes et al., 2020). This versatility in testing variants of the same protein might be useful to overcome some of the limitations discussed above.

Another limiting factor for the inducible systems is the application of the inducer. Whereas the current experimental setup demonstrates the suitability of the TetON system in axenic culture, *Ustilago maydis* is a biotrophic fungus where specific research questions might require an inducible system during the infection. Therefore we tested the suitability to add Tet to maize infected with fluorescent protein reporter strains under the control of the Tet promoter. We could detect by confocal microscopy Tet-dependent expression of the transgene mCherry and eGFP 24 hours post induction in 10 days old maize seedlings, three days post-infection. Nevertheless, we observed also interference with the infection frequency of maize seedlings. In Tet-treated condition, in some areas of the infected leaves, we observed that hyphae were not able to expand although expressing the transgene. Likely Tet- concentrations within the plant body vary and optimization of Tet concentrations and ways of application into the plant is required. It will be a challenge to ensure equal concentrations of the inducer within the whole plant which might lead to various levels of expression in the infected host and variation of side-effects.

Furthermore, as Tet acts as a potent antibiotic, its application in complex interspecies interactions might influence the composition of the microbiome and interfere directly or indirectly with specific research questions, a fact that needs to be considered while designing the respective experiments.

Another limitation the Tet system bears for use in a complex plant-fungal interaction setup is, that it is not possible to ensure an even Tet concentration to the fungal hyphae which are growing intra- and intercellularly inside the maize plant. Therefore, quantitative differences need to be interpreted with care as the accessibility of the inducer to the target cells might not be equal within the tissue. Nevertheless in multicellular organisms like plants, a Tet-operated system with a chimeric transactivator was already employed for the expression of plant genes (Weinmann et al., 1994).

Although *U. maydis* is the so far best-studied smut fungus among its ~1300 smut relatives, still more than 40% of its genes are annotated as “unknown” or “hypothetical”. The now available tet-regulated ‘TetOFF’ system (Zarnack et al., 2006) and ‘TetON’ system (developed in the current study) for *U. maydis* will be an additional tool for researchers to elucidate gene functions in this important model pathogen.

## Data availability statement

The original contributions presented in the study are included in the article/**Supplementary Material**. Further inquiries can be directed to the corresponding author.

## Author contributions

AD conceived the research. KI, SU and AD designed the research. SU generated the GreenGate DNA modules used in this study. CG helped in cloning. AD helped in experimental designing and supervised the experiments. KI and NN performed the experiments and analyzed the data. All authors contributed to the article and approved the submitted version.

## Funding

The research leading to these results received funding from the European Research Council under the European Union’s Seventh Framework Programme ERC-2013-STG (grant agreement: 335691), the Austrian Science Fund (I 3033-B22), the Austrian Academy of Sciences, and the Deutsche Forschungsgemeinschaft (DFG, German Research Foundation) under Germany's Excellence Strategy EXC-2070-390732324 (PhenoRob) and DFG grant (DJ 64/5-1).

## Acknowledgments

We would like to thank the GMI/IMBA/IMP core facilities for their excellent technical support. We would like to acknowledge Dr. Sinéad A. O’Sullivan from DZNE, University of Bonn for providing anti-GFP antibodies. The authors are thankful to the Excellence University of Bonn for providing infrastructure and instrumentation facilities at the INRES-Plant Pathology department.

## Conflict of Interest

The authors declare that the research was conducted in the absence of any commercial or financial relationships that could be construed as a potential conflict of interest.

## Publisher's note

All claims expressed in this article are solely those of the authors and do not necessarily represent those of their affiliated organizations, or those of the publisher, the editors and the reviewers. Any product that may be evaluated in this article, or claim that may be made by its manufacturer, is not guaranteed or endorsed by the publisher.
