# Supplementary material for: Tetracycline-controlled (TetON) gene expression system for the smut fungus Ustilago maydis
Source: Front Fungal Biol. 2022 Oct 19;3:1029114. doi: 10.3389/ffunb.2022.1029114 (PMC10512375; doi:10.3389/ffunb.2022.1029114)
Supplement: Supplementary file 2 [file DataSheet_1.docx]

**Supplementary Material:**


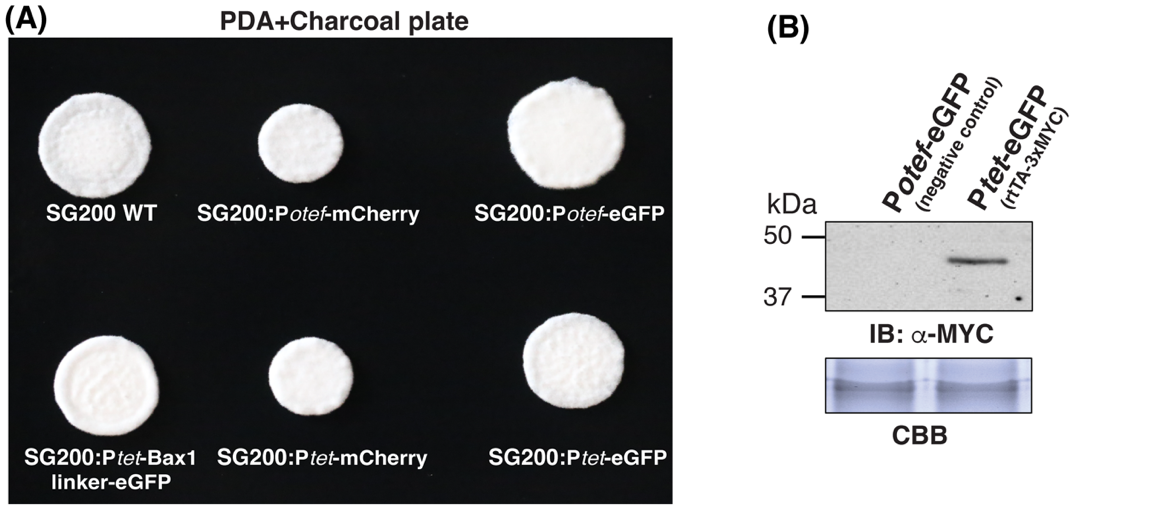


***Supplementary Fig 1***. ***(A)*** *Filamentation of U. maydis strains on PDA-Charcoal plate. The image was captured 48 hours post spotting.* ***(B)*** *Immunoblot with anti-MYC antibodies on total protein extracts to detect expression of rtTA-3xMYC (~42 kDa). Coomassie brilliant blue (CBB) stained gel is shown as a loading control.*

*
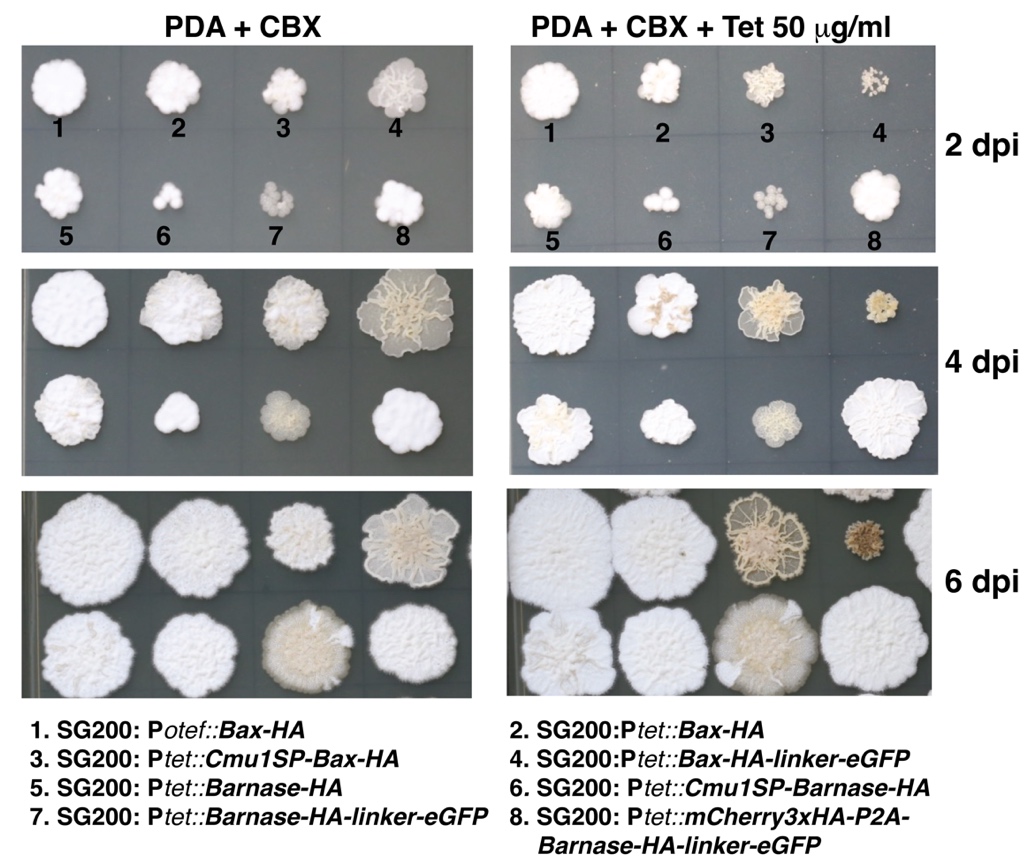
*

***Supplementary Fig 2.*** *Testing of Bax1 and Barnase toxicity in U. maydis with different constructs. Different constructs of Bax1 and Barnase were tested for their toxicity upon expression in U. maydis. Whereas Bax1 shows as a fusion protein with eGFP a growth inhibitory effect upon Tet induction, Barnase constructs tested seemed to be tolerated or not functionally expressed in U. maydis.*


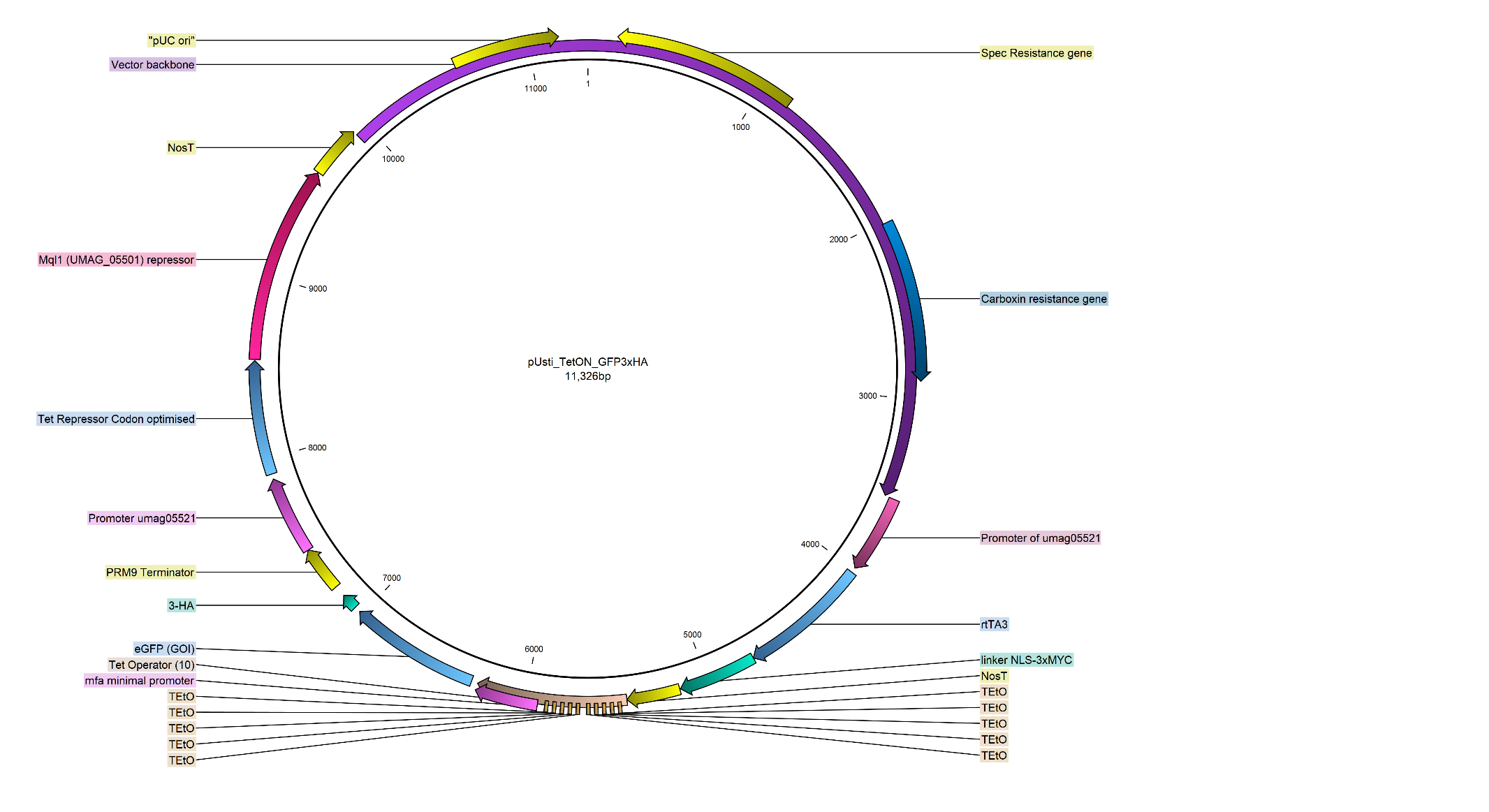


***Supplementary Fig 3****. Vector map of p123-P_tet_-eGFP-3xHA.*


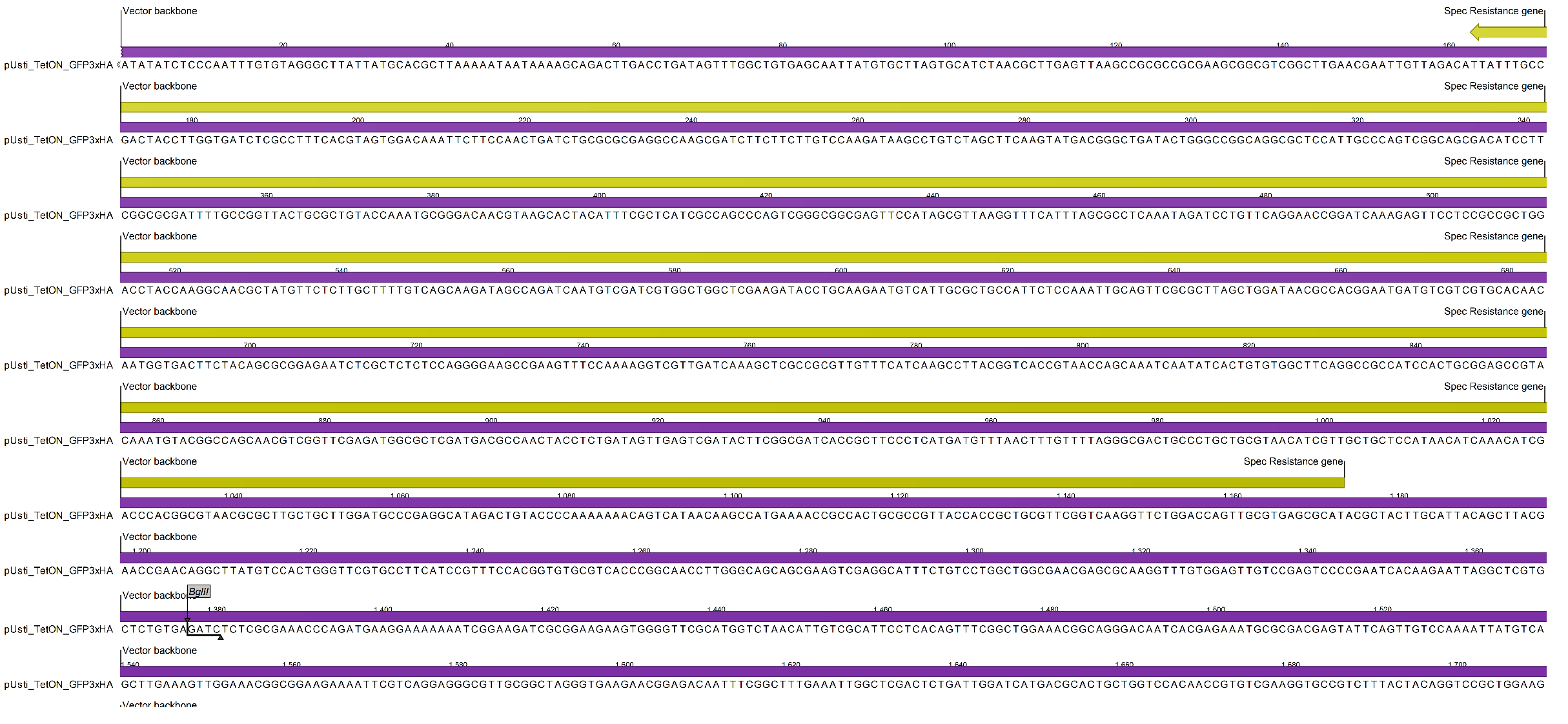


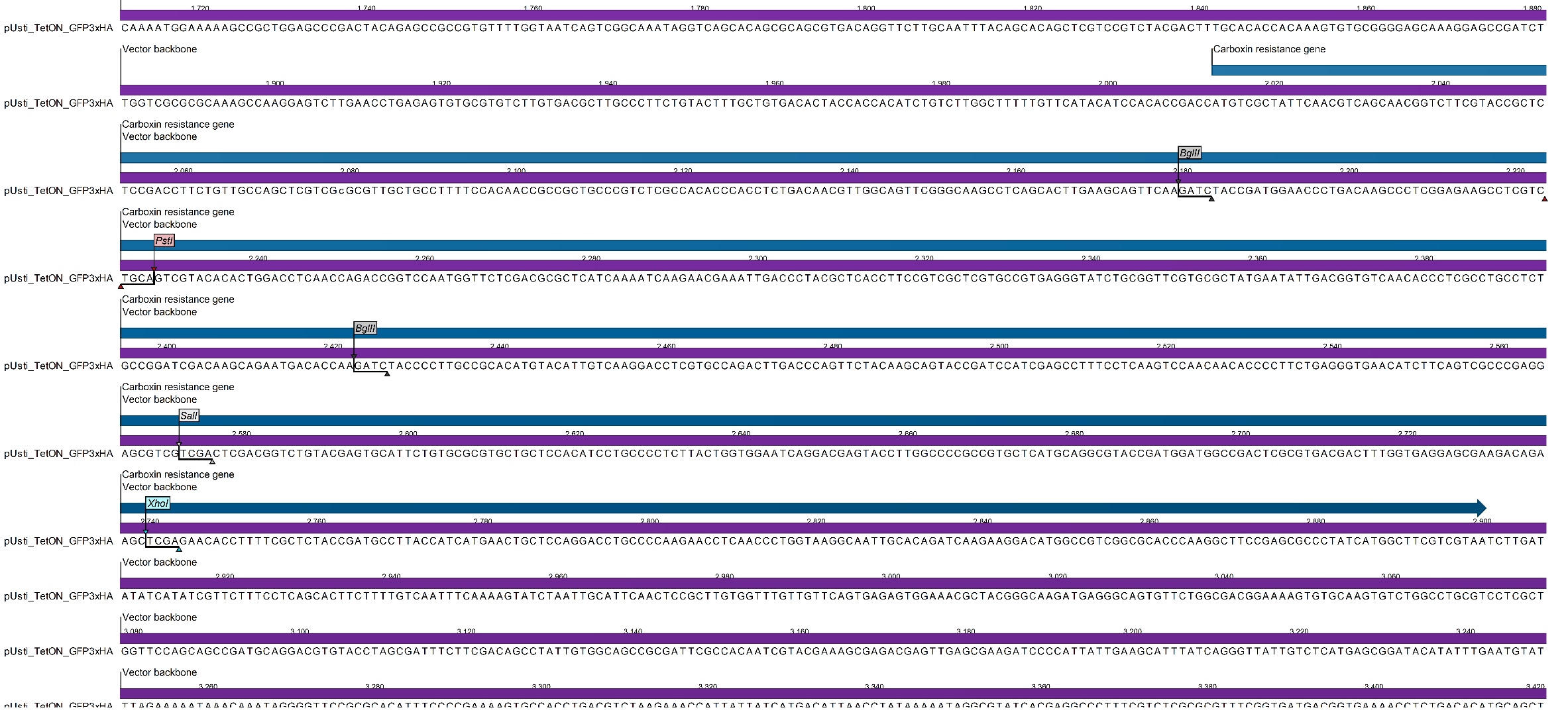


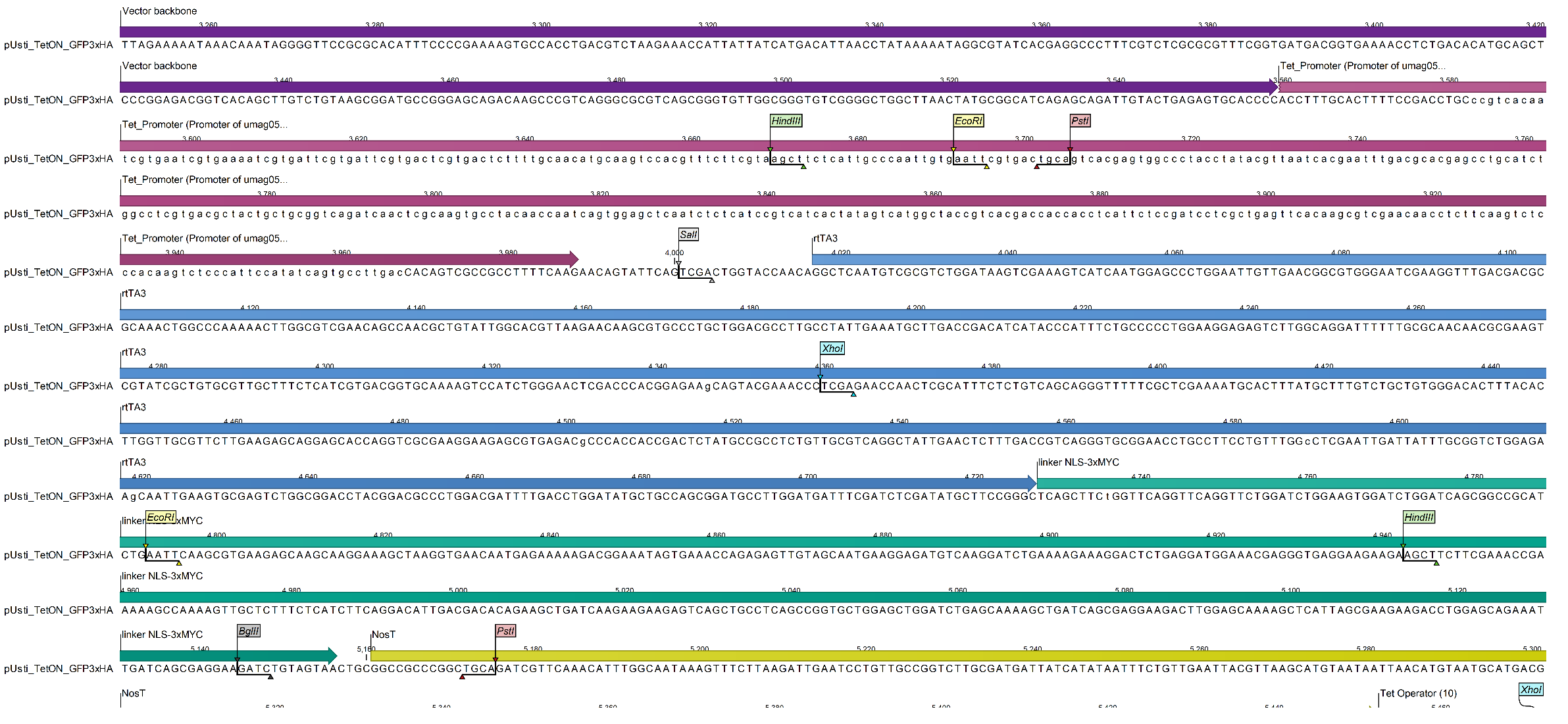


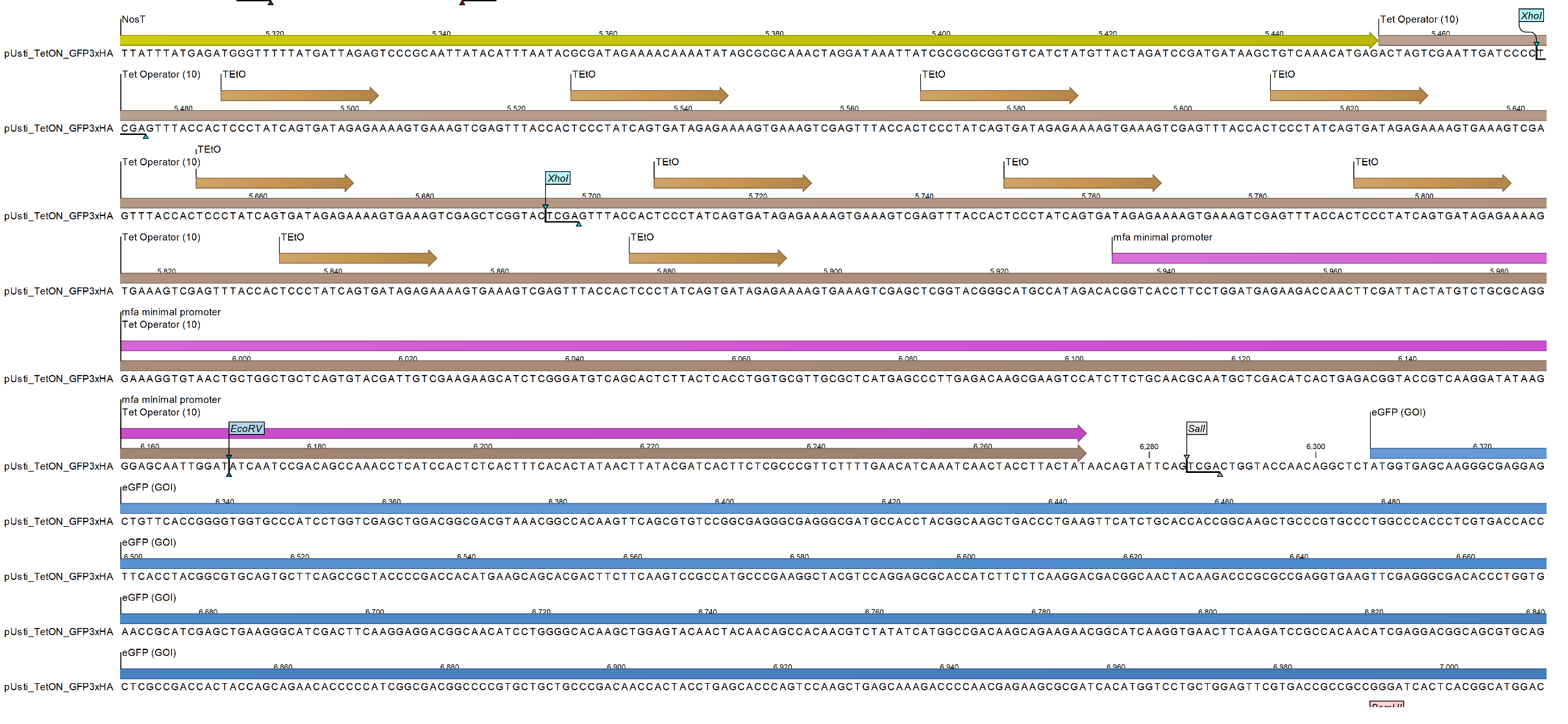


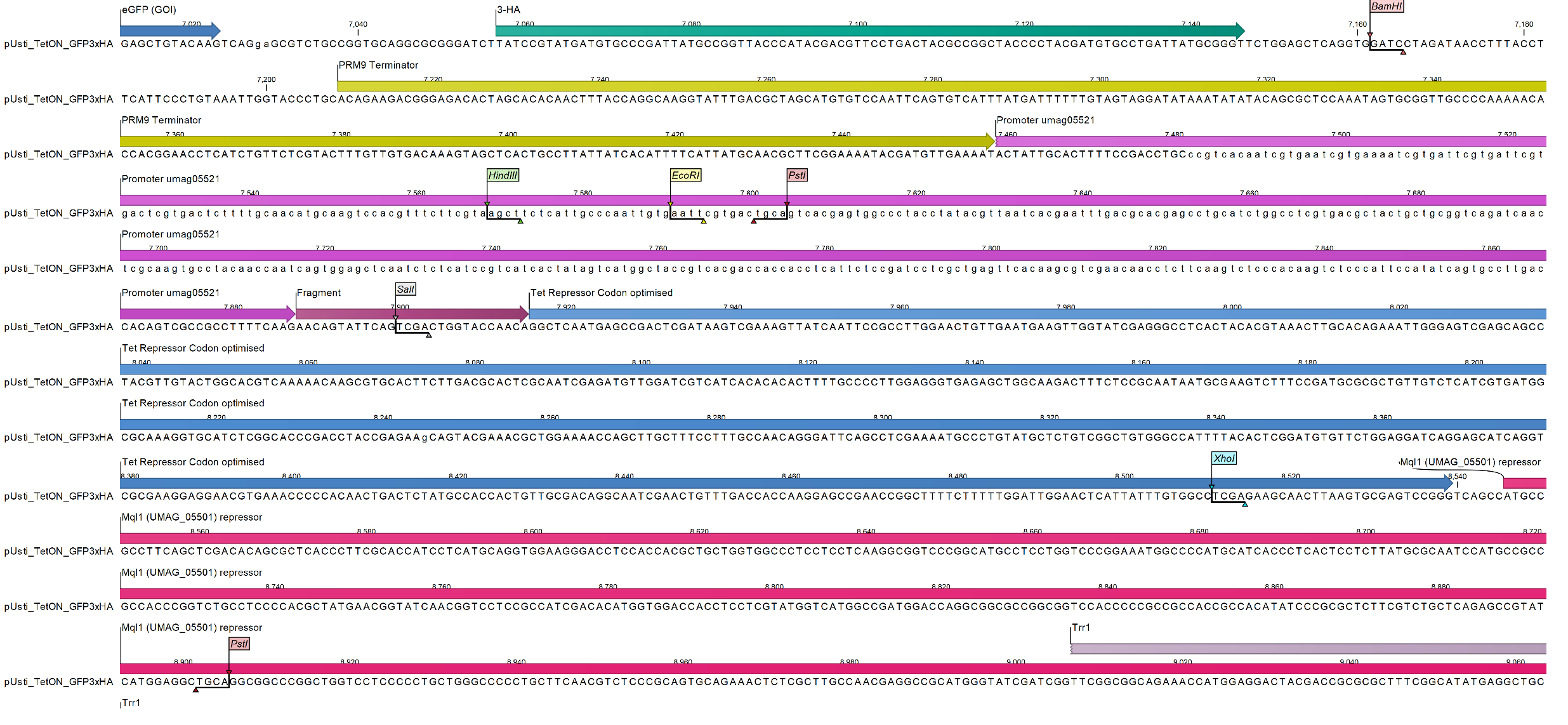


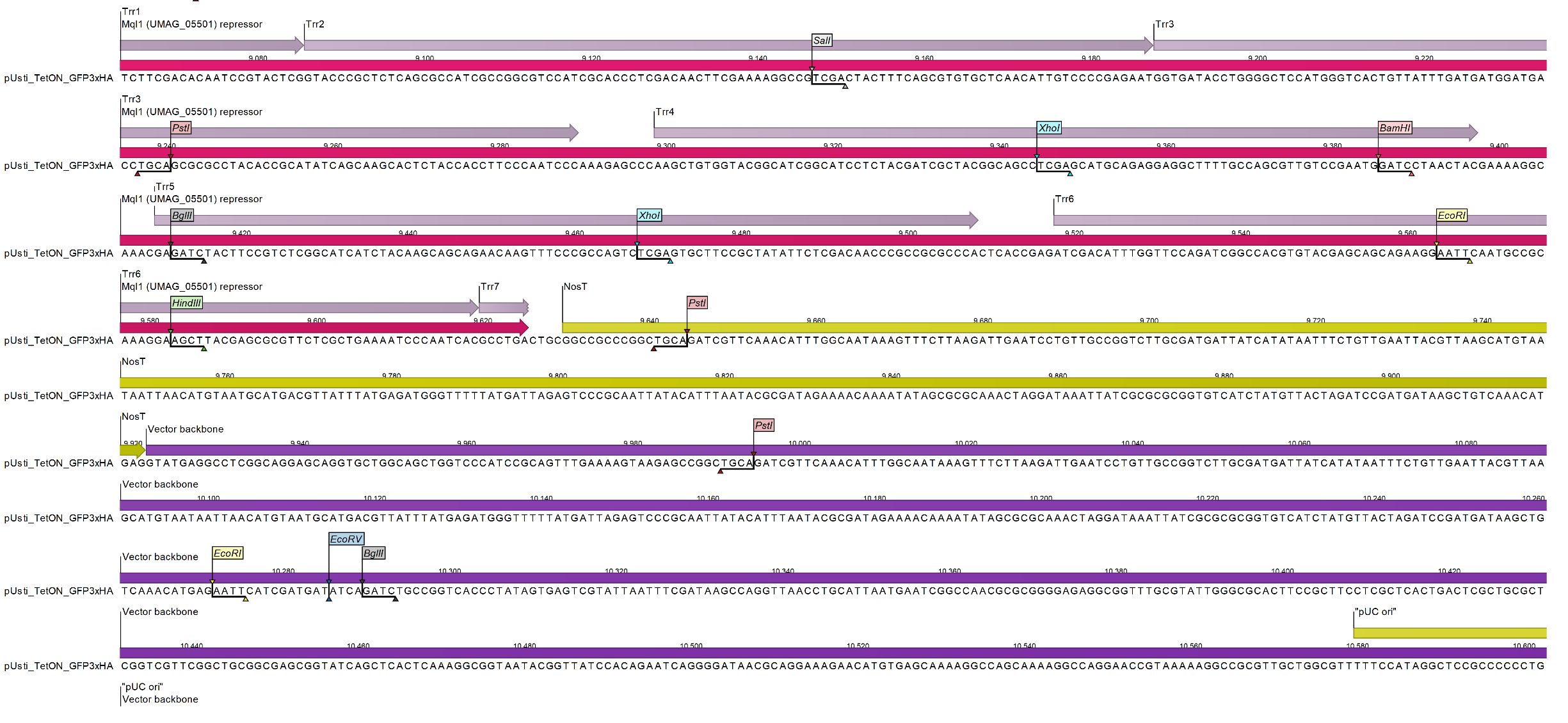


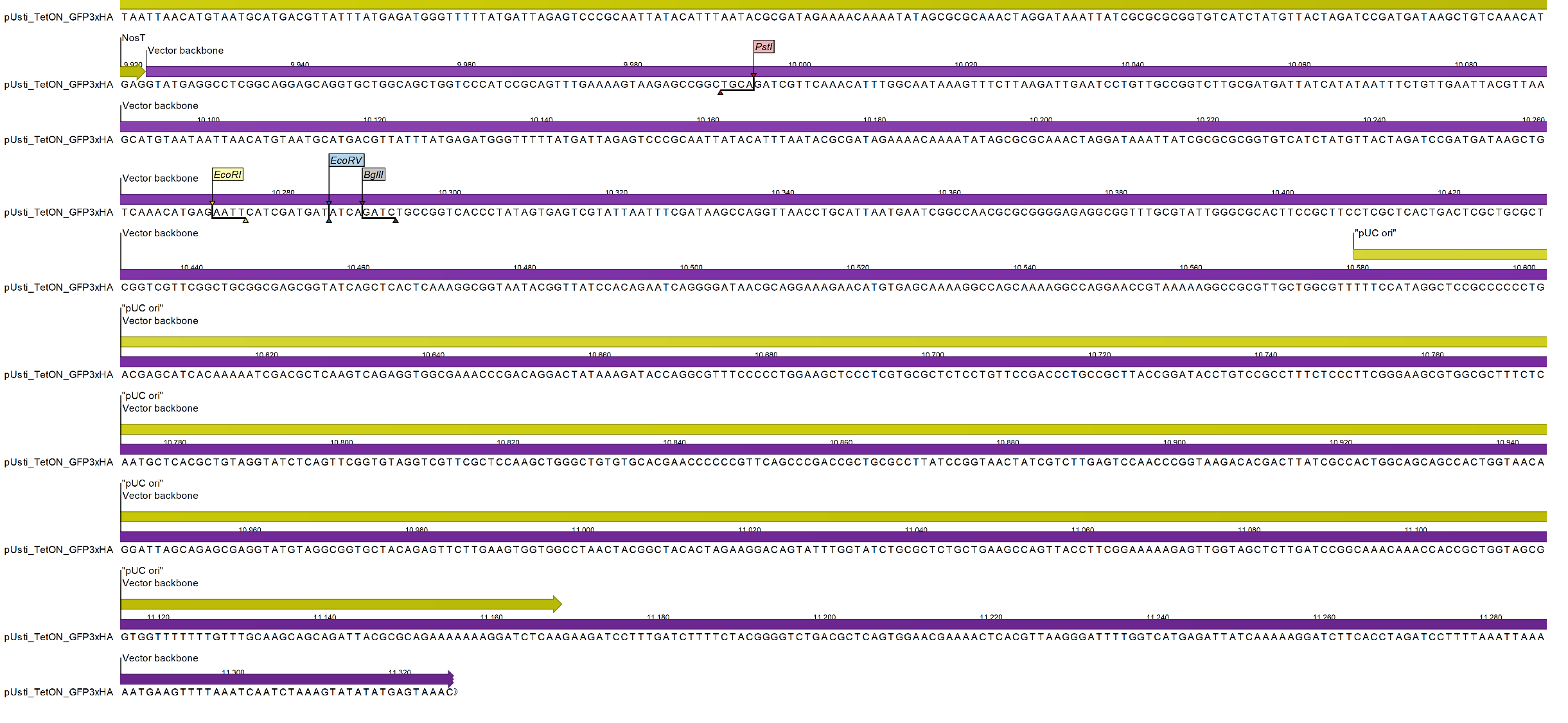


***Supplementary Fig 4.*** *Annotated plasmid DNA map of p123**-P_tet_-eGFP-3xHA vector depicting positions and sizes of all DNA elements needed for TetON regulation in U. maydis.*

**Supplementary Table 1: List of plasmids used in this study**

| No | Plasmid name | Used in |
| --- | --- | --- |
| 1 | *p*123-P*_otef_*-mCherry-3xHA | Fig 2,3,4,5 |
| 2 | *p*123-P*_otef_*-eGFP-3xHA | Fig 2,3,4,5 |
| 3 | *p*123-P*_tet_*-mCherry-3xHA | Fig 2,3,4,5 |
| 4 | *p*123-P*_tet_*-eGFP-3xHA | Fig 2,3,4,5 |
| 5 | *p*123-P*_tet_*-Bax1HA-linker-eGFP | Fig 3, 4 |
| 6 | *p*123-P*_otef_*-Bax1-HA | Supplementary Fig 2 |
| 7 | *p*123-P*_tet_*-Bax1-HA | Supplementary Fig 2 |
| 8 | *p*123-P*_tet_*-Cmu1SP-Bax1-HA | Supplementary Fig 2 |
| 9 | *p*123-P*_tet_*-Barnase-HA | Supplementary Fig 2 |
| 10 | *p*123-P*_tet_*-Cmu1SP-Barnase-HA | Supplementary Fig 2 |
| 11 | *p*123-P*_tet_*-Barnase-HA-linker-eGFP | Supplementary Fig 2 |
| 12. | *p*123-P*_tet_*-mCherry-3xHA-P2A-Barnase-HA-linker-eGFP | Supplementary Fig 2 |

**Supplementary Table 2: List of primers used in this study and their purposes**

| **Primer Name** | **Primer sequence (5'-3')** | **Used for** |
| --- | --- | --- |
| GG-Bax-HA_F | ATATGGTCTCAGGCTCTATGGACGGCTCGGGCGAG | Cloning of Bax-HA |
| GG-Bax-HA_R | ATATGGTCTCTCTGAAGCGTAATCTGGAACATCG |  |
| ProMed_05521_F | ATGGTCTCAACCTTTGCACTTTTCCGACCTGC | Cloning of promoter of UMAG_05521 from *U. maydis* |
| ProMed_05521_R | ATGGTCTCATGTTCTTGAAAAGGCGGCGACTGTG |  |
| rtTA3-C_F | ATATGGTCTCAGGCTCAATGTCGCGTCTGGATAAGTC | Cloning of rtTA3 transcription factor |
| rtTA3-D_R | TATAGGTCTCACTGAGCCCGGAAGCATATCGAGATC |  |
| P9141_NLS_D_F | ATATGGTCTCATCAGCTTCTGGTTCAGGTTCAGGTTC | Cloning of NLS with 3xMYC |
| P7473_3xmyc_R-E | TATAGGTCTCTGCAGTTACTACAGATC |  |
| TetR-C_F | ATATGGTCTCAGGCTCAATGAGCCGACTCGATAAGTC | Cloning of TetR* codon optimized for *U. maydis* |
| TetR-D_R | TATAGGTCTCACTGACCCGGACTCGCACTTAAGTTG |  |
| Ssn6_Repressor_Ssn6-ATG_F | ATATGGTCTCATCAGCCATGCCGCCTTCAGCTCGACAC | Cloning of Ssn6 Repressor ortholog from *U. maydis* (Mql1) fragment 1 |
| Ssn6_Repressor_Ssn6F1_R | ATGGTCTCACGAACCGATCGATACCCATGCGGCCTCGTTGGC |  |
| Ssn6_Repressor_Ssn6F2_F | ATGGTCTCATTCGGCGGCAGAAACCATGGAGGACTACGACCG | Cloning of Ssn6 Repressor ortholog from *U. maydis* (Mql1) fragment 2 |
| Ssn6_Repressor_Ssn6-359_R | ATATGGTCTCAGCAGGGCGTGATTGGGATTTTCAG |  |
